# Optimization of the viability PCR for accurate detection of *Staphylococcus aureus* in food samples

**DOI:** 10.1371/journal.pone.0324819

**Published:** 2025-05-23

**Authors:** Mai Dinh Thanh, Gemma Agustí, Anneluise Mader, Francesc Codony

**Affiliations:** 1 Department of Biology, Chemistry and Pharmacy, Freie Universität Berlin, Berlin, Germany; 2 Technical Department, Reactivos para Diagnóstico, S.L., Polígono Industrial Mas d’En Cisa, Sentmenat, Barcelona, Spain; 3 Department Safety in the Food Chain, German Federal Institute for Risk Assessment (BfR), Berlin, Germany; 4 Department Microbiology, Laboratori d’Aigües de Mataró, Mataró, Spain; Universidad Autonoma de Chihuahua, MEXICO

## Abstract

*Staphylococcus* (*S.*) *aureus* is a prominent foodborne pathogen that can cause food poisoning due to its staphylococcal toxins. Controlling the viable levels of *S. aureus* is crucial for ensuring food safety. The detection of *S. aureus* during routine quality control is still primarily conducted using traditional culture-based methods, which are time-consuming and unable to detect viable but non-culturable cells. Viability PCR (vPCR) – a combination of traditional (or quantitative) PCR with photo-reactive DNA-intercalating dye(s) – has been introduced as an alternative to detect viable cells by excluding those with compromised membranes using molecular methods. Despite the success of the vPCR methodology, avoiding false-positive results remains a significant challenge. To enhance the accuracy of vPCR results for *S. aureus*, several approaches have been proposed by various researchers in the past decade; however, complete PCR signal suppression of dead cells has not been achieved. In this study, we developed a strategy to detect only viable *S. aureus* cells by combining double PMA treatment with a low PMA concentration and performing a tube change between the last dark incubation and light exposure to improve the vPCR protocol. For pure cultures, the optimized protocol was able to completely suppress DNA signals from 5.0 × 10^7^ dead cells in a final reaction volume of 200 µl. For artificially contaminated food samples with such a high dead cell count, complete PCR signal reduction was observed in ground pepper, - oregano, and infant milk powder, while ground paprika, - allspice, and - pork exhibited PCR signals close to the detection limit. To simulate conditions in real samples, we artificially contaminated ground paprika, - pork, and milk powder with a low number of viable cells (~1.9 cfu/ml) and a high number of heat-inactivated *S. aureus* (~4.8 × 10⁶ cells/ml). The results showed that the optimized protocol is effective in detecting only the desired live cells, even in the presence of a high dead cell count. Our findings highlight that vPCR can be an accurate and reliable method with strong potential for high-throughput detection of live *S. aureus* cells in certain food matrices.

## 1. Introduction

*Staphylococcus aureus* (*S. aureus*) is a Gram-positive, non-spore forming, facultative human pathogenic bacterium. Approximately 30% of the human population carries *S. aureus* as a nasal commensal [1]. However, *S. aureus* is also known to cause severe skin- and soft tissue infections [2] as well as staphylococcal food poisoning [3]. Food poisoning caused by ingestion of heat-stable staphylococcal enterotoxins is among the leading causes of foodborne outbreaks in the European Union [4]. Frequently positive tested foods include raw milk, dairy- and meat products. In condiments, *S. aureus* has rarely been detected [5,6]. However, *S. aureus* holds the potential to survive for months in low moisture foods due to its tolerance towards low a_w_ levels [7,8]. Thus, contaminated condiments might serve as a vehicle for transferring *S. aureus* to foods with optimal conditions for growth and toxin production.

In order to maintain high food safety standards, routine testing is highly relevant for the entire food production process, as well as for public authorities. For the assessment of microbial contamination and related hygienic standards, there are governmental regulations and recommendations. Depending on the specific food or food group, the guidance and critical values may vary. In the EU, food safety and process hygiene criteria are defined by the European Commission. For example: At the end of the manufacturing process, the concentration of coagulase-positive staphylococci in milk powder should not exceed a limit of 100 cfu/g. The analytical reference methods are the ISO 6888 series. The ISO 6888–1 [9] and -2 [10] standards are for direct plate count, while ISO 6888–3 [11] is a method for enumeration of low levels and requires sample pre-enrichment before colony isolation. ISO 6888–3 is also used to examine the presence or absence of *S. aureus* in a single tube. Additionally, if samples indicate values over 10^5^ cfu/g, the batch has to be tested for staphylococcal enterotoxin [12].

The culture-based method is considered the ideal approach for detecting microorganisms. However, it has several limitations and drawbacks, including inhibition of detection by certain matrices due to antimicrobial effects (e.g., cloves, oregano [13]) or high microbial background levels, significant waste generated from duplicates and dilutions, as well as being labor-intensive and time-consuming. For instance, detection including confirmation of coagulase-positive staphylococci can take up to four days (ISO 6888–1); if enrichment is required, this could take up to two days longer (ISO 6888–3). Moreover, viable but non-culturable (VBNC) cells remain undetectable. These microorganisms are viable but cannot be cultured or grown under conventional laboratory conditions because they exist in a dormant state with low metabolic activity. The VBNC state is commonly adopted by microorganisms as a survival strategy in response to unfavorable or stressful conditions. Despite being non-culturable, VBNC cells can retain the potential to reactivate under certain environmental conditions, regaining their ability to be cultured, reactivating their metabolic functions, and restoring their capacity to cause infections and diseases, thus posing a significant threat to public health [14,15].

Polymerase chain reaction (PCR) provides an alternative approach for microorganism detection across various matrices. This rapid and highly sensitive method enables specific identification of target microorganisms through DNA amplification. The main drawback of the classical PCR is that it cannot differentiate between viable and dead populations or extracellular DNA, leading in an overestimation of actual viable cell count [16,17].

To overcome this issue, viability PCR (vPCR) has been introduced to exclude free DNA and DNA from cells with compromised membranes [18,19], which are considered to be dead cells. The concept of vPCR is quite simple: By adding a photoactive DNA-intercalating dye such as ethidium monoazide (EMA) or propidium monoazide (PMA) to the sample prior to the DNA extraction, the dye enters the compromised cells and intercalates into the DNA. The samples are then exposed to bright visible light to photo-activate the dye. Photolysis converts the azide group of the dye into a highly reactive nitrene radical, which forms a non-reversible covalent bond with the DNA, rendering it inaccessible to the polymerase. As a result, DNA from compromised cells is excluded from amplification during the PCR [20].

vPCR has been applied to detect a wide spectrum of various food relevant microorganisms including *S. aureus* [21,22]. However, vPCR also has its own set of advantages, challenges and limitations, as demonstrated in many studies, which are summarized in detailed reviews by Fittipaldi et al. and Codony et al. In summary, the success of vPCR depends on several key factors such as dye type and concentration, dye incubation conditions (temperature and time), light source and photoactivation time, microorganism and matrix type, as well as PCR amplification conditions (e.g., amplificon length, temperature and time) [23,24]. The key limitation of the vPCR method lies in the fact that differentiation between viable and dead cells is based solely on membrane integrity. As PMA can only penetrate cells with damaged membranes, cell monitoring after disinfection using methods that do not directly affect membrane permeability (e.g., UV treatment) is not possible [25–27]. Due to the strong dependency of vPCR on cell membrane status, VBNC cells can be detected, but not “ghost cells” [20].

Despite numerous efforts in recent years to optimize vPCR protocols for these critical factors (e.g., applying reagent enhancers, using a dye mix, changing reaction tubes, double photo-activation or double dye treatment), residual PCR signals from dead cells are still commonly observed, especially in samples with high dead cell count [24,28]. However, in some cases, complete signal reduction at high dead cell concentrations could be achieved by applying a combination of different approaches [29,30]. Although available optimization protocols have successfully reduced the PCR signal of *S. aureus* to a significant extent, complete PCR signal suppression of dead cell population has not yet been achieved [31–35]. Consequently, these protocols would still overestimate the actual cell count of viable cells and thus are not reliable for routine quality control testing, especially when evaluating the effectiveness of decontamination.

The aim of our study is to optimize vPCR for the detection of viable *S. aureus* despite high dead cell concentrations (up to 10^7^ cfu/ml). By combining different approaches, we developed a robust vPCR protocol capable of exclusively detecting live *S. aureus* in pure cultures. In a simulation, we artificially contaminated ground paprika, - pork, and milk powder with a low number of viable (~2 cfu/ml) and a high number of heat-inactivated (~10^6^ cells/ml) *S. aureus* cells and could demonstrate that the optimized protocol is capable of detecting only the desired live cells.

## 2. Materials and methods

The section 2.1 ‘General methods descriptions’ provides detailed descriptions of the materials and methods used, without referring to any experiments. For descriptions of a particular experiment (experiment design), please refer to section 2.2 ‘Experiments descriptions’.

### 2.1. General methods descriptions

#### 2.1.1. Bacterial strain and stock culture conditions.

Three *S. aureus* strains were used in this study. Two of them were obtained from the American Type Culture Collection: a) strain ATCC 6538 (isolated from a human lesion, no enterotoxin production, able to form biofilm); b) strain ATCC 9144 (unknown origin, positive PCR for enterotoxin G and I as well as Panton Valentine Leucocidine). The third strain – SA 56302/14 – was obtained from a local laboratory; this strain was isolated from a food sample and was not further characterized.

All strains were kept frozen as glycerol-stocks at -20 °C. To obtain single colonies, the frozen isolate was transferred to Plate Count Agar (PCA) plates (Liofilchem, Roseto degli Abruzzi, IT). After 24 h of growth at 37 °C, the agar plates were kept at 4 °C during the experiment setup.

#### 2.1.2. Food matrices.

Dried and ground culinary spices and herbs – paprika (*Capsicum annuum*), pepper (*Piper nigrum*), allspice (*Pimenta dioica*) and oregano (*Origanum vulgare*) – were provided by FUCHS Gewürze GmbH (Dissen, Germany). The matrices were sealed in aroma-tight food packaging bags, each containing 500 g. The bags were stored at room temperature (23 ± 1 °C) and stayed sealed until use.

Ground pork meat and infant milk powder were obtained from a local supermarket in Terrassa. Ground pork meat was stored at 4 °C until use.

#### 2.1.3. Bacteria suspension production.

To obtain a working bacterial suspension, a single colony was streaked onto a PCA plate and incubated at 37 ºC for 14–16 h. A loop of the bacterial lawn was then harvested from the PCA plate and suspended in 10 ml phosphate buffered saline (PBS, pH 7.4). The cell density was adjusted to an optical density (OD_600_) of 0.35, corresponding to 2.0 × 10^8^ cfu/ml to 5.0 × 10^8^ cfu/ml. Actual cell counts are provided at the respective experiment. Unless otherwise stated, cell counts were determined by plating a serial dilution of the bacterial suspension on PCA plates.

#### 2.1.4. Dead cells production.

To obtain heat killed cells, aliquots of 1 ml cell suspension was heated at 80 °C for 20 min using a standard laboratory heat block (thermomixer comfort, Eppendorf, Hamburg, Germany) at 900 rpm. The loss of cell viability was verified by plating 100 μl of the cell suspension on PCA plates, followed by incubation at 37 ºC for 24 h.

#### 2.1.5. PMA treatment.

PMA dye (GenIUL, Terrassa, Spain), was dissolved in PCR grade water (VWR, Llinàs del Vallés, Spain) to obtain a 2 mM stock solution, which was stored at -20 °C until needed.

All reactions were conducted at a final volume of 200 µl. If necessary, PBS was added to reach the 200 µl reaction volume. Low-binding reaction tubes (GenIUL) were used for all experiments.

**Double PMA treatment**: PMA was added to reach the respective target concentrations. Samples were incubated in the dark (dark incubation) at room temperature (RT) for the specified duration (either 1 or 5 min) to allow dye penetration into the cells with damaged membranes. Photo-induced crosslinking of PMA (photo-activation) was achieved by exposing the samples to 5 min of blue light using an LED-based device, the PhAST Blue instrument (GenIUL), at 100% intensity. Afterwards, the second PMA treatment was performed by adding PMA to the same reaction tube at the same concentration as the first treatment, followed by dark incubation for the same duration as before. The samples were then transferred to new reaction tubes (GenIUL) and were subsequently exposed to 5 min of light using the PhAST Blue instrument at 100% intensity.

**Single PMA treatment:** In the case of a single PMA treatment, samples were treated with the desired PMA concentration only once. After adding the required volume of PMA, samples were incubated in the dark at RT for a specified period. Following the dark incubation, the samples were transferred to new reaction tubes and were subsequently exposed to 5 min of light using the PhAST Blue instrument at 100% intensity.

After a single or double PMA treatment, samples were centrifuged at 12,300 × g (micro centrifuge Microstar 12, VWR, Radnor, USA) for 5 min and supernatants were discarded.

#### 2.1.6. DNA extraction and real-time PCR assay.

DNA was extracted using the v-DNA reagent and v-DNA buffer (GenIUL), which is based on alkaline lysis, in the absence of chaotropic agents, but with a combination of resins that remove different organic molecules and metallic ions, which may affect the quality of the DNA. Briefly, cell pellets were resuspended in 200 μl of v-DNA reagent and were vortexed at 3,200 rpm (MPS-1 Multi Plate Shaker, bioSan, Riga, Latvia) for 5 min. Then, the cells were incubated at 80 ºC for 10 min at 1,200 rpm in a heat block. After incubation, 600 µl of v-DNA buffer was added and vortexed again at 3,200 rpm for 2 min. The samples were subsequently centrifuged at 7,500 × g for 2 min and 100 µl of the supernatant containing the DNA was transferred to a new tube.

Real-time PCR amplifications were performed using the PikoReal™ Real-Time PCR System (Thermo Fisher Scientific, Barcelona, Spain) with the following protocol: an initial denaturation at 95 °C for 12 min, followed by 45 cycles of 10 s at 95 °C, 10 s at 62 °C and 30 s at 72 °C, concluding with a melting curve analysis (72–88 °C). Each reaction contained 4 μl of 5X HOT FIREPol Evagreen® qPCR Mix Plus (Solis BioDyne, Tartu, Estonia), 0.4 μM of each forward and reverse primers, and 5 μl of DNA template (final volume: 20 µl). PCR-grade water was used as a non-template control.

The primers (Nuc_fw: 5′-TCA GCA AAT GCA TCA CAA ACA G-3′, Nuc_rw: 5′-CGT AAA TGC ACT TGC TTC AGG-3′) according to Poulsen et al. [36] targeted a 255 bp fragment of the *S. aureus* specific *nuc* gene, which encodes an extracellular thermostable nuclease.

#### 2.1.7. Amplification efficiency.

The real-time PCR efficiency was determined using the slope of a standard curve, which was generated from 10-fold serial dilutions of *S. aureus* DNA extracted from a pure culture with a known concentration. To obtain the standard curve, cycle threshold (C_t_) values were plotted against the corresponding log_10_ cell count. The amplification efficiency (E) was calculated by using the equation: E = 10^-1/slope^ – 1 [12].

#### 2.1.8. Statistical analysis.

Statistical analyses were conducted using SPSS Statistics 30 (IBM, Armonk, USA). Comparisons of means between two or more groups were performed using one-way univariate analysis of variance (ANOVA). For multiple comparisons, Tukey’s HSD post-hoc test was applied to evaluate differences between groups. Differences were considered statistically significant at P < 0.05.

### 2.2. Experiments descriptions

All experiments were conducted three times independently.

#### 2.2.1. Optimization of PMA treatment using pure culture.

To determine the optimal PMA concentration and treatment procedure, live and dead cells of *S. aureus* strain ATCC 6538 (3.6 × 10^7^ ± 1.1 × 10^7^ cells in a final reaction volume of 200 µl) were subjected to either a single treatment with 0 µM (untreated), 1 µM, 10 µM, 25 µM, 50 µM or 100 µM PMA or a double treatment with 1 µM, 10 µM or 25 µM PMA. The dark incubation time was set to 1 min, while the light exposure time was 5 min.

To find out the optimal PMA dark incubation time, additional live and dead sample aliquots were subjected to double treatments with 10 µM or 25 µM PMA, combined with dark incubation times of 5 min or 15 min. The light exposure time remained at 5 min.

The PMA concentration and dark incubation time combination that yielded the best results was then tested on two additional *S. aureus* strains – ATCC 9144 (4.6 × 10^7^ ± 2.0 × 10^7^ cfu/200 µl) and SA 56302/14 (3.2 × 10^7^ ± 5.6 × 10^6^ cfu/200 µl) – to evaluate the robustness of the optimized method.

#### 2.2.2. Efficacy of PMA treatment for live- and dead cells in pure culture.

Based on *S. aureus* ATCC 6538 suspensions with 5.0 × 10^8^ ± 1.4 × 10^8^ cfu/ml, dead cells and a ten-fold serial dilution of live cells, ranging from 10^7^ to 10^2^ cfu/ml, were prepared. The serial dilutions of live cells (10^7^ to 10^2^ cfu) were mixed with a constant number of 10^7^ dead cells in a final reaction volume of 200 µl. These samples were then subjected to double PMA treatment with either 0 µM or 10 µM PMA. As controls, samples containing either only 5.0 × 10^7^ live cells or dead cells were processed under the same conditions.

To assess the linearity of the results, the calculated PCR efficiency was compared with that of the standard curves. Since cell loss or DNA loss may occur during treatment and DNA extraction processes, two standard curves were generated. The first standard curve was created using a serial dilution made of pre-extracted DNA (no independent experiment repetition, but there are 2 replicates for real-time PCR values) to evaluate the actual PCR efficiency. To determine the DNA extraction efficiency, the second standard curve was generated using a serial dilution of untreated live cells ranging from 2.1 × 10^7^ ± 7.7 × 10^6^ to 2.1 × 10^2^ ± 7.7 × 10^1^ cfu/200 µl reaction volume.

#### 2.2.3. vPCR efficiency in food samples.

Ground spices and herbs (allspice, paprika, pepper and oregano), ground pork meat and infant milk powder were suspended in peptone water (PW) (Oxoid, Basingstoke, UK); spices and herbs at a ratio of 1:20 (w/v), meat and infant milk powder at a ratio of 1:10 (w/v). A 1 g portion of each matrix was suspended and allowed to settle for 5 min. Then, 100 µl of the supernatant of each sample was transferred into a new reaction tube and artificially contaminated with 100 µl of either live or dead *S. aureus* ATCC 6538 cells (5.4 × 10^7^ ± 7.7 × 10^6^ cells/200 µl). To remove rough particles, samples were centrifuged at 67 × g for 2 min and the supernatants were transferred into new reaction tubes, followed by centrifugation at 12,300 × g for 5 min. The resulting pellets were washed with 500 µl PBS, resuspended in 200 µl PBS and transferred into new reaction tubes. Live-cell samples were subjected to double PMA treatment with either 0 µM or 10 µM PMA; dead-cell samples were subjected to double PMA treatment with 10 µM PMA. PW served as a control matrix and was treated under the same conditions.

#### 2.2.4. Detection of live cells in the presence of dead cells in food samples.

Paprika powder, ground pork meat and infant milk powder were used for the detection of live *S. aureus* ATCC 6538 cells in the presence of dead *S. aureus* cells. For meat and milk powder samples, 25 g were mixed with 225 ml of PW in a filter stomacher bag (IUL S.A., Barcelona, Spain). For paprika samples, 10 g were mixed with 240 ml PW (a higher dilution ratio is required to avoid a mousse-like consistency). Then, 1 ml of live cells (4.8 × 10^2^ ± 5.8 × 10^1^ cfu/ml) and 2.5 ml of dead cells (4.8 × 10^8^ ± 5.8 × 10^7^ cells/ml) were inoculated to each suspension and homogenized for 30 s in a stomacher (Maxicator, IUL, Barcelona, Spain). 250 ml of PW inoculated with bacteria served as a control. Contaminated samples were incubated at 37 °C. At 0-, 24- and 48-hours post-incubation, 120 µl of supernatants were taken for *S. aureus* detection.

The mixtures were centrifuged at 47 × g for 2 min, to remove rough particles. Then, 100 µl of the supernatant were transferred to a new reaction tube and centrifuged at 12,300 × g for 5 min. The cell pellets were suspended in 500 µl of PBS and centrifuged again at 12,300 × g for 5 min. After discarding the supernatant, the pellet was suspended in 200 µl PBS and transferred to a new tube. Samples were then subjected to double PMA treatment with either 0 µM or 10 µM PMA.

In addition to the vPCR, live *S. aureus* cells were also detected using Baird-Parker agar plates according to ISO 6888–1 standard as a culture-based control.

## 3. Results

### 3.1. Optimization of PMA treatment using pure culture

**Fig 1:**
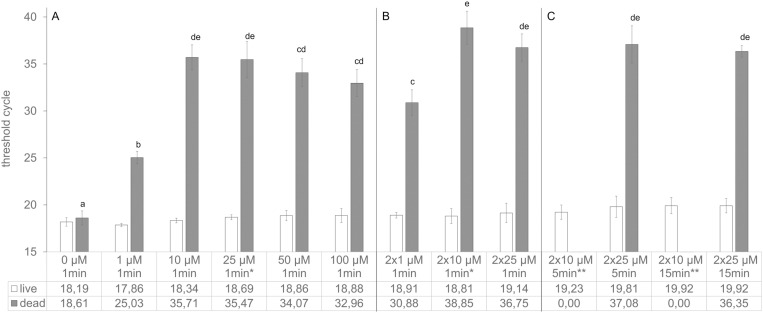
Effect of different PMA concentrations, treatment procedures and dark incubation times on live and dead *S. aureus* cells. Live or dead *S. aureus* ATCC 6538 cells (3.6 × 10^7^ cells/200 µl sample) were single treated (part A) or double treated (part B and C) with varying PMA concentrations, in combination with dark incubation times of 1 min (part A and B) or 5 or 15 min (part C). Means and standard deviations (n = 3) are depicted. Significant differences (P < 0.05) between dead cell samples are indicated as small letters (a-e). No statistical differences were found between live cell samples. * no PCR signal in one of 3 samples. ** no PCR signal in all three samples, therefore, means are stated as ‘0.00’.

To determine the optimal PMA treatment conditions, three key factors – concentration, treatment procedure (single or double treatment) and dark incubation time – were investigated using *S. aureus* ATCC 6538. The results are summarized in Fig 1.

The difference in threshold cycle (∆C_t_) between PMA-treated and -untreated dead cells was 6.4 cycles at 1 µM PMA and increased to 17.1 cycles at 10 µM, indicating a dose-response effect. Higher concentrations (25–100 µM) did not further enhance PCR signal reduction. A slight decline in efficacy was noted above 25 µM, although Tukey-HSD analysis indicated no significant differences (Fig 1A).

Double PMA treatment (Fig 1B) increased ∆C_t_ values compared to a single treatment (Fig 1 A), with the largest difference observed at 1 µM PMA (5.9 cycles). At higher concentrations, the difference flattened as C_t_ values approached the limit of detection (LOD). Accordingly, no statistically significant differences between single and double treatment were found at 10 µM and 25 µM PMA.

For the tests shown in Fig 1, parts A and B, the dark incubation time was set to 1 min, where differences in ∆C_t_ for concentration and treatment procedure were most distinct. The tests depicted in Fig 1, part C aimed to determine the optimal dark incubation time, testing 5 and 15 min with double PMA treatment using 10 µM or 25 µM PMA. With 25 µM PMA, no complete signal reduction for dead cells was achieved at any incubation time, but the detected C_t_ values were close to the LOD. However, double PMA treatment with 10 µM PMA and 5 or 15 min of dark incubation time reduced PCR signals below the LOD.

The effect on viable cells was evaluated by comparing the ∆C_t_ values between the PMA-treated and -untreated live cells. All tests in parts A to C showed ∆C_t_ values below 1.7 cycles, with parts A and B reaching a maximum of 0.9, and part C showing values of at least 1. This result suggests that longer PMA exposure times affect live cells more strongly than higher PMA concentrations (up to 100 µM) with shorter incubation times. However, multiple comparisons using the Tukey-HSD post-hoc test revealed no significant differences between C_t_ means of all live cell samples. Thus, the optimal treatment identified in this series was the combination of double PMA treatment with 10 µM PMA and 5 min of dark incubation time for each dye treatment step.

Two additional *S. aureus* strains – ATCC 9144 and SA 56302/14 – were tested using the optimal PMA treatment. The results, shown in Table 1, indicate similar outcomes for these two strains compared to the ATCC 6538 strain: PCR signals of treated dead cell samples were successfully suppressed below the LOD. In this experiment, C_t_ of treated live cell samples were slightly higher – but statistically significant – compared to untreated live cell samples.

**Table 1:** Effect of double PMA treatment on live and dead cells of two different *S. aureus* strains.

	ATCC 9144	SA 56302/14
Live cells without treatment	18.55 ± 0.04^a^	20.93 ± 0.65^a^
Live cells with PMA treatment	19.9 ± 0.32^b^	22.65 ± 0.45^b^
Dead cells with PMA treatment	no signal	no signal

Live and dead cells of *S. aureus* strains ATCC 9144 (4.6 × 10^7^ cells/200 µl sample) and SA 56302/14 (3.2 × 10^7^ cells/ 200 µl sample) were subjected to double PMA treatment with 10 µM PMA and 5 min dark incubation, followed by real-time PCR detection. Mean threshold cycle values and standard deviations (n = 3) are presented. Different subscript letters indicate significant differences between treated and untreated live cell samples (P < 0.05).

In summary, double PMA treatment with 10 µM PMA, combined with a 5-minutes dark incubation and a 5-minutes light exposure time, resulted in PCR signal suppression below the LOD for 10^7^ dead cells in 200 µl final reaction volume across all three tested *S. aureus* strains, with only a slight impact on live cells. Therefore, this combination was selected as the optimal treatment and used for all subsequent experiments.

### 3.2. Efficacy of PMA treatment on live and dead cells in pure cultures

**Fig 2:**
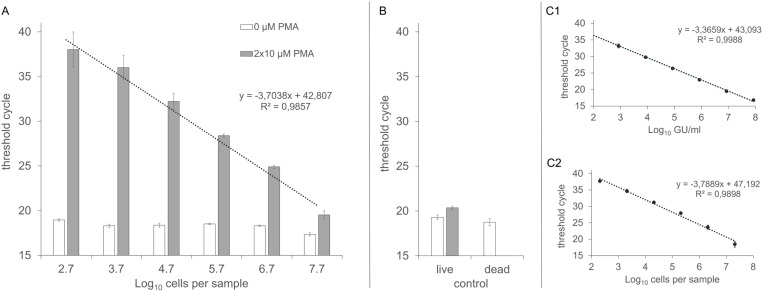
Effect of double PMA treatment on serial dilutions of live *S. aureus* cells in the presence of dead cells. Serial dilutions of live *S. aureus* ATCC 6538 cells (5.0 × 10^7^ to 5.0 × 10^2^ cfu) in the presence of 5.0 × 10^7^ dead *S. aureus* cells in a final reaction volume of 200 µl were subjected to double treatment with either 0 µM (white bars) or 10 µM (grey bars) PMA, followed by real-time PCR (part A). Control samples containing either 5.0 × 10^7^ live or dead cells (populations were not mixed) were shown in part B. No PCR signal was detected in any replicates of the PMA-treated dead cell samples, therefore no respective bar is shown in the graph. Standard curves are depicted in part C1 (serial dilutions made from extracted DNA) and part C2 (DNA extracted from a serial dilution of live cells). Means, standard deviations (n = 3), the linear regression equation and R^2^ values are provided.

The efficacy of the optimized vPCR protocol – double PMA treatment with 10 µM PMA, combined with 5 min dark incubation and 5 min light exposure time – was tested on serial dilutions of live *S. aureus* cells (5.0 × 10^7^ to 5.0 × 10² cfu), each mixed with 5.0 × 10^7^ dead cells. A linear regression analysis was performed by plotting C_t_ values against the respective log₁₀ live *S. aureus* cell counts (Fig 2A).

The mixed populations without PMA (Fig 2A) showed mean C_t_ of 18.3 ± 0.5, which were comparable to the controls (Fig 2B), yielding mean C_t_ of 19.3 ± 0.3 for live cells without PMA, 18.8 ± 0.2 for dead cells without PMA, and 20.4 ± 0.4 for live cells with PMA. Treated dead cells in the controls showed no PCR signals, confirming reliable suppression below the LOD.

Despite the high concentration of dead cells, the C_t_ values with PMA exhibited a strong linear correlation with the live cell concentration (R² = 0.9857). PCR efficiency in the mixed population was 86.2%, compared to 98.2% using pure DNA (Fig 2, C1) and 83.6% for untreated live cells (Fig 2, C2). These results indicate that PMA treatment effectively eliminated signals from 5.0 × 10^7^ dead *S. aureus* cells per reaction, with only a minor reduction in PCR efficiency, likely due to inhibitory residues from the DNA extraction kit.

The LOD was 5.0 × 10² cells per 200 µl final reaction volume (2.5 × 10³ cfu/ml; yet this value can be adjusted by concentration through centrifugation, therefore values are expressed per reaction). Given the DNA extraction method (800 µl final DNA eluate) and the DNA template volume used (5 µl), each PCR reaction contained approximately 3 gene copies. At this gene copy level, the mean C_t_ was 38.02 ± 1.97. A further tenfold serial dilution would not yield reliable or accurate PCR signals. Therefore, C_t_ values above 40 should be considered below the practical detection limit. Occasional C_t_ values greater than 40 should be confirmed by melting curve analysis to exclude false-positive results.

### 3.3. vPCR efficiency in food samples

**Fig 3:**
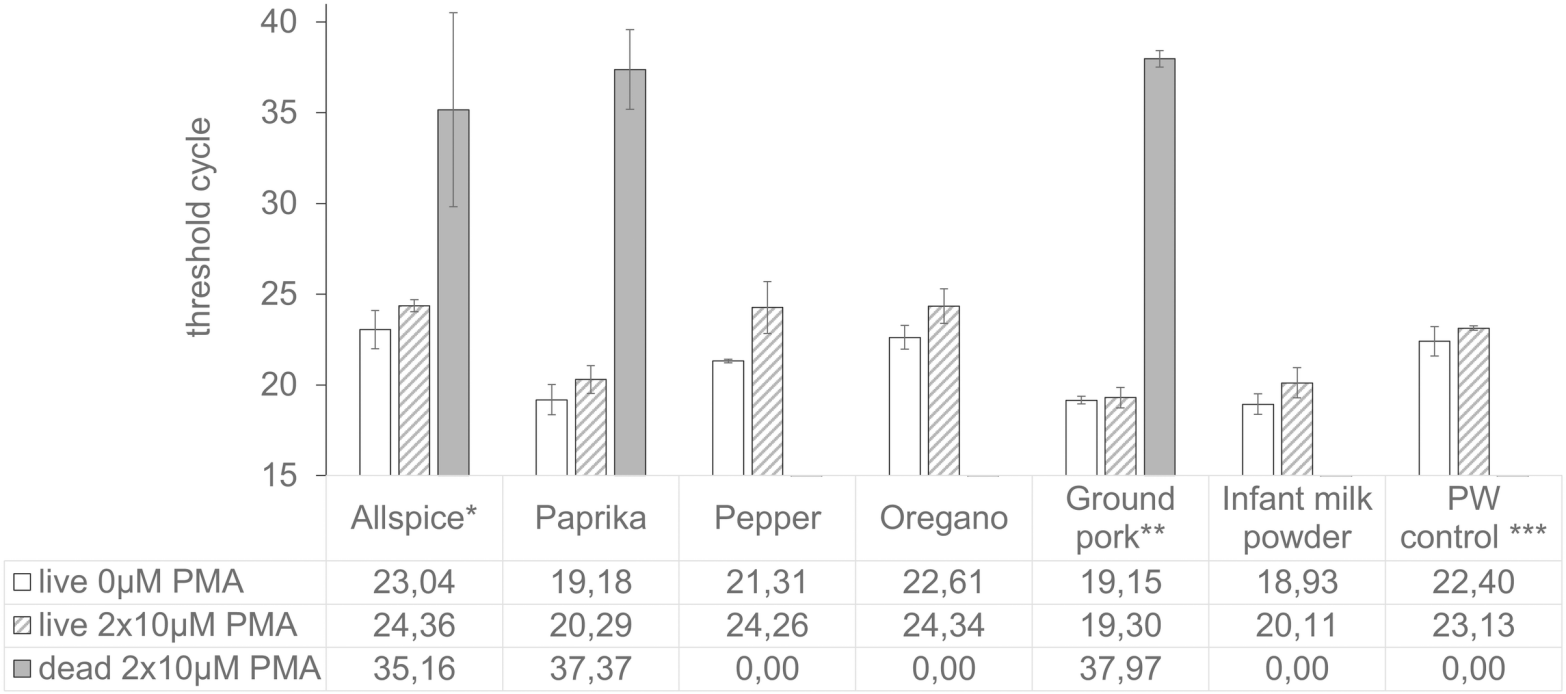
Effect of double PMA treatment on live or dead *S. aureus* cells in food samples. Supernatants from various foods were artificially contaminated with live and dead *S. aureus* ATCC 6538 cells (5.4 × 10^7^ cfu/200 µl sample) and subjected to double PMA treatment with either 0 µM (white bars: live cells) or 10 µM (shaded bars: live cells; grey bars: dead cells) PMA, followed by real-time PCR. Mean C_t_ values and standard deviations (n = 3) are depicted. * One sample exhibited a C_t_ signal of 41,23; two other samples showed C_t_ signal between 31 and 33. ** No PCR signal was detected in one of three treated samples containing dead cells. *** In the PW control, 2 samples showed no PCR signal, 1 sample exhibited a C_t_ signal of 39,99 with an unspecific melting peak, thus, this result was interpreted as 0.00 (no signal).

Supernatants from six food suspensions – allspice, paprika, pepper, oregano, ground pork meat, and infant milk powder – and PW as a control were artificially contaminated with 5.4 × 10^7^ live or dead *S. aureus* cells per sample. Samples were subjected to double PMA treatment or left untreated to evaluate the applicability and efficiency of the optimized vPCR protocol in food matrices (Fig 3).

In dead cell samples, PCR signals were completely suppressed to levels below the LOD for pepper, oregano, and infant milk powder, while allspice, paprika, and ground pork samples exhibited strongly reduced PCR signals close to the LOD. Notably, one out of three meat samples showed no signal, and allspice showed a C_t_ of 41.23. In PW controls, two out of three treated samples showed no signal; the third showed an unspecific melting peak (76.1 °C), outside the expected range (78.5–78.9 °C), and was interpreted as ‘no signal’.

Treated live cell samples showed slightly higher C_t_ values than untreated, consistent with prior results. Among untreated live cell samples, only paprika, ground pork, and infant milk powder fell within the expected range (C_t_ 18–20). Other spice samples, including the PW control, exhibited C_t_ values approximately 3 cycles higher than expected, suggesting approximately 1 log₁₀ cell loss during washing.

In summary, the optimized vPCR protocol effectively eliminates PCR signals from dead *S. aureus* cells in several food matrices to levels below the LOD, with minimal impact on live cells. However, its efficacy is reduced in complex matrices such as paprika powder, allspice powder, and minced meat.

### 3.4. Detection of live cells in the presence of dead cells in food samples

This follow-up experiment aimed to evaluate the accuracy of this vPCR protocol for mixed populations in food matrices under realistic conditions, and its efficiency in reducing the PCR signal at a lower dead cell concentration compared to the previous experiment.

Paprika (1:25 w/v), ground pork (1:10 w/v), and infant milk powder (1:10 w/v) were artificially contaminated with a mixed *S. aureus* population (1.9 cfu/ml live cells and 4.8 × 10⁶ cells/ml dead cells), and incubated at 37 °C for 48 h. Due to differences in the dilution ratios, the *S. aureus* concentration per gram of dry matrix corresponded to 48 cfu/g live cells and 1.2 × 10⁸ dead cells/g for paprika, and 19 cfu/g live cells and 4.8 × 10^7^ dead cells/g for ground pork and infant milk powder. The results are summarized in Table 2.

**Table 2:** Detection of live *S. aureus* ATCC 6538 cells in artificially contaminated food samples (containing both live and dead cells) using the vPCR method.

Time	0h		24h			48h	
Treatment	0 µM PMA	2x10 µM PMA	0 µM PMA	2x10 µM PMA	cfu/ml	0 µM PMA	2x10 µM PMA
Sample							
**Paprika**	25,99 ± 0,25	No signal	26,42 ± 0,58	37,23 ± 0,83	5.3 × 10^2^ ± 6.4 × 10^2^	19,67 ± 0,6	20,88 ± 0,32
**Ground pork**	28,28 ± 0,14	No signal	27,66 ± 1,18	29 ± 1,04	5.7 × 10^5^ ± 4.0 × 10^5^	19,98 ± 0,81	20,75 ± 0,57
**Infant milk** **powder**	27,46 ± 0,39	No signal	28,23 ± 0,67	34,09 ± 1,88	6.0 × 10^4^ ± 2.2 × 10^4^	18,72 ± 0,85	19,4 ± 1,1
**PW control**	32,27 ± 0,54	No signal	26,4 ± 0,93	28,12 ± 0,86	>1.0 × 10^6^	18,86 ± 1,65	22,56 ± 0,5

Paprika, ground pork, infant milk powder, and PW control samples were artificially contaminated with a mixed population of live and dead *S. aureus* cells and enriched at 37 °C for 48 h. Aliquots (100 µl each) were collected at 0-, 24-, and 48 h for cultural detection using Baird Parker plates and molecular detection using vPCR. Plate counts at 0 h were below the LOD (10 cfu/ml) and results at 48 h were above 1.0 × 10⁶ cfu/ml for all samples. Means and standard deviations (n = 3) are presented in the table.

Immediately after spiking, untreated samples showed C_t_ values between 25.99 ± 0.25 and 28.28 ± 0.14 (expected C_t_ ~ 25), whereas PMA-treated samples showed no PCR signals, which is consistent with the negative plate counts. The PW control exhibited a higher C_t_ (32.27 ± 0.54), likely due to cell loss during washing steps.

After 24 h of enrichment, untreated samples exhibited C_t_ values similar to those at point in time 0 h. In contrast, PMA-treated samples produced positive PCR signals reflecting viable cell growth, with C_t_ values consistent with the corresponding plate counts for each matrix, except for the PW control. In the PW control, C_t_ values (28.12 ± 0.86) were approximately 1 log₁₀ lower than expected, again suggesting cell loss during washing. At this point in time, the live cell populations in food samples increased, but remained below the initial dead cell count, whereas in the PW control, live counts exceeded the inoculated dead cell load. The presence of autochthonous microbiota in paprika and ground pork, along with the use of non-selective enrichment medium, likely slowed *S. aureus* growth compared to the PW control. Additionally, *S. aureus* colony identification on Baird Parker plates was challenging due to microbial background interference.

After 48 h, both untreated and PMA-treated samples showed C_t_ values between 18.72 ± 0.85 and 20.88 ± 0.32, indicating that the live cells had surpassed the initial dead cell population.

In summary, the optimized vPCR protocol was able to correctly detect the live cell count in the presence of 10⁶ dead cells/ml across tested food matrices, at least on a qualitative level. The washing steps applied during sample preparation prior to PMA treatment – which were not the focus of our study – need further optimization to prevent cell loss.

## 4. Discussion and conclusion

### 4.1. vPCR protocol improvement

vPCR is theoretically a rapid, sensitive, and reliable method for detecting viable cells by excluding membrane-compromised cells (a conservative definition of dead cells). However, practical experience has raised concerns about its effectiveness, particularly due to the incomplete suppression of PCR signals, which can lead to false-positive results – especially when there is a high background of dead cells. This limitation has hindered its widespread use in routine quality control. Consequently, several efforts have been made to improve the ability of vPCR to specifically detect live cells.

EMA has been shown to be less selective than PMA, particularly for *S. aureus*. Nocker et al. [19] demonstrated visually that EMA can enter live *S. aureus* cells, whereas PMA does not affect live cells. EMA is based on ethidium bromide (which carries one positive charge) and an azide group, while PMA is based on propidium iodide (which carries two positive charges) coupled with an azide group. The azide group is responsible for covalent binding to DNA. The higher positive charge on PMA likely contributes to its greater impermeability through intact cell membranes [19], which is why we use PMA for our vPCR protocol.

The efficacy of PMA is based on membrane integrity: intact membranes in viable cells act as a barrier to the dye, whereas PMA can penetrate dead cells with damaged membranes and crosslink with their DNA upon light activation. Since some killing methods (e.g., UV radiation) do not directly affect cell membranes, heat treatment is commonly used to obtain dead bacterial cells for demonstrating vPCR potential [19,31]. For *S. aureus*, Zhang et al. [37] evaluated various heat treatment conditions and proposed 80 °C for 20 min as the optimal condition to kill cells without releasing DNA. We adopted the same heat-treatment condition to produce dead cells with destabilized membranes, and heat treatment at 80 °C for 20 min successfully inactivated the cells without detectable DNA release (as evidenced by similar PCR signals between live and dead cells in Fig 1).

One key factor for the success of PMA treatment is the PMA concentration and exposure time. PMA shows a dose-response effect, which was demonstrated by applying a tenfold serial dilution of PMA (ranging from 0.05 µM to 50 µM) to 10^7^ cells/ml dead *Salmonella* [38]. Many optimization approaches have involved concentrations between 1 µM and 50 µM, sometimes up to 100 µM [33–35,39–41]. In our study, we tested various PMA concentrations (1–100 µM) under different treatment procedures and dark incubation times to determine the optimal conditions.

With a single treatment, PMA concentrations of 10 µM and 25 µM were most effective in suppressing the PCR signals from 3.6 × 10^7^ dead cells, yielding ~4.6 log₁₀ genome unit (GU) reduction. Increasing the PMA concentration up to 100 µM did not further suppress the PCR signal from dead cells. These findings are consistent with Chang and Lin [32], who reported 3.1–3.8 log₁₀ reduction using 2.4–37.3 µM PMA under comparable conditions (albeit with 5 min dark incubation and 20 min light exposure using a 500-W halogen lamp), and no additional suppression at higher PMA concentrations. Chang and Lin also systematically evaluated varying PMA concentrations (2.4, 3.7, 16.2, and 37.3 µM) on serial dilutions of live or dead cells (ranging from 10³ to 10^7^ cfu/ml), finding that complete suppression of the PCR signal from dead cells was achievable only up to 10⁵ cells/ml. For higher dead cell counts, the signal could be substantially reduced but not completely neutralized [32].

Complete signal reduction for high dead cell counts by single PMA treatment (16.2 µM PMA with 5 min dark incubation and 5 min light exposure using a 500-W halogen lamp) was reported by Zhang et al. [37], although their use of traditional PCR (agarose gel) resulted in a relatively high LOD (10⁴ cfu/ml). In contrast, real-time PCR typically achieves an LOD of 10² cfu/ml, making it unclear whether dead cell DNA was completely neutralized under those conditions.

At low PMA concentrations, as shown by Codony et al. [38], a linear dependency exists between PMA concentration and suppression efficiency. However, it seems like beyond a certain concentration, a plateau near the LOD is reached – especially in the presence of a high background of dead cells – where further increases in PMA concentration do not yield additional suppression [32,35,39,41]. In our study with *S. aureus*, this plateau began at approximately 10 µM PMA. With the standard protocol (single treatment with 5 min dark incubation followed by 5–15 min of light exposure), none of the tested conditions completely suppressed the DNA signal from high dead cell counts below the LOD.

Regarding live cell samples, our data showed that single PMA treatment had only a moderate effect on treated live cells – resulting in C_t_ values roughly 1 cycle higher than untreated live cells. This slight increase may be partly due to a naturally occurring fraction of compromised or dead cells in the population. This notion is supported by studies such as Stewart et al. [42] on aging and death of *E. coli* and Yáñez et al. [43] on the effects of PMA on exponentially growing *Legionella pneumophila*. In the latter study, qPCR results without PMA pre-treatment yielded approximately one log₁₀ higher cell counts than plate counts, whereas qPCR coupled with PMA pre-treatment produced results similar to those obtained by plating.

Kobayashi et al. [44] reported that a concentration of 50 µM PMA caused the C_t_ value of live *S. aureus* cells (~10⁵ cfu/ml) to drop by around 2 cycles. Chang and Lin [32] observed a similarly low effect for high cell counts (10⁸ cfu/ml), however, a significantly stronger effect was observed for lower live cell counts (10⁶ cfu/ml and below) when PMA concentrations of 16.2 µM or higher were used. They proposed 2.4 or 3.7 µM PMA as the optimal concentration for suppressing dead cell DNA amplification while minimally affecting live cell signals. In contrast, for species such as *E. coli*, *Salmonella*, and *Listeria monocytogenes*, high PMA concentrations did not significantly impact live cell counts [39,41,45]. These findings underscore the importance of optimizing PMA concentration on a species-specific basis to avoid underestimation of live cells and overestimation of dead cells.

The main challenge when using PMA is enhancing its permeability to dead cells to maximize PCR signal reduction [23]. To address this, many optimization approaches have incorporated membrane-destabilizing agents. However, for *S. aureus*, none of the protocols combining a detergent or surfactant with a single PMA treatment fully eliminated the PCR signal from dead cells [33,34]. For instance, Zi et al. [34] reported that in dead cells, the ∆C_t_ between samples treated with Triton X-100 and those treated with PBS alongside 10 µM PMA was only 1.5 cycles, whereas the ∆C_t_ between treated and untreated live cells was 4 cycles. In our opinion, this trade-off is too substantial to be considered effective.

For species like *Salmonella* and *Listeria monocytogenes*, where high PMA concentrations do not significantly affect live cell counts, combining high PMA concentrations with tube changes and double light treatment seems promising. For *Salmonella* (using a dye blend), complete signal reduction was achieved in 3 of 4 samples containing 10^7^ dead cells/ml [30]. For *Listeria monocytogenes*, the new protocol resulted in a ∆C_t_ of 3.7 cycles compared to the traditional protocol [29]. Since a high PMA concentration of 50 µM is not optimal for *S. aureus*, double light treatment might not be appropriate. Thus, we considered other optimization approaches to increase PMA efficiency in suppressing dead cell DNA:

a) Double PMA incubation: Pan and Breidt [45] applied double PMA incubation (each with 50 µM PMA, 5 min dark and 5 min light incubation) on Listeria *monocytogenes*, observing an average C_t_ increase of 2.8 cycles compared to single treatment. We therefore applied similar conditions but with a lower concentration (10 µM) to enhance DNA neutralization while minimizing the impact on live cells.
b) Tube change: A microtube change between dark incubation and light exposure has been shown to further reduce amplification signals. For instance, an additional reduction of ~1.64 log₁₀ (~5 C_t_) was reported for *Salmonella* [46], while a small but statistically significant reduction of 1.6 C_t_ was observed for *Listeria monocytogenes* [29]. The increased C_t_ values may be due to free DNA adhering to the tube walls [47], making it inaccessible to PMA. However, during the subsequent extraction procedure, the DNA is released and can be amplified during PCR [46]. In our protocol, we adopt a tube-change approach after the second PMA incubation.
c) Light source: Since PMA’s absorption maximum is 464 nm, blue light-emitting devices are more effective than halogen lamps, which also generate heat that can interfere with the process [23,48]. For standardization of the photoactivation step, we used a commercial blue LED-based instrument.
d) Amplicon length: To enhance vPCR effectiveness, we incorporated findings by Li and Chen [28] and Martin et al. [49]. They tested various primers resulting in amplicon lengths ranging from 65 to 260 bp and 75–417 bp, respectively, and demonstrated that longer amplicons led to greater PCR signal suppression in PMA-treated samples compared to shorter ones. Therefore, we chose a primer pair that produces an amplicon of 255 bp [36].

By combining a double treatment with 10 µM PMA (each with 5 min dark incubation and 5 min light exposure) and a tube change between the last dark and light exposure, we found an optimal approach to suppress the PCR signal from 10^7^ dead cells to below the LOD (10² cells/sample). Interestingly, double treatment with 25 µM PMA did not produce the same result, regardless of whether the incubation time was 5 or 15 min. The reason might be due to the short light inactivation time, as a sufficient light dose is required for complete photolysis of PMA. Codony et al. [38] calculated that for 50 µM PMA, an exposure time of at least 15 min is needed. Our protocol’s 5-minutes exposure might be too short to inactivate all PMA molecules. Nocker et al. [19] also noted that with increasing PMA concentration, the light exposure time needs to be increased to promote complete photolysis. This could also explain why the efficiency in reducing the PCR signal from dead cells starts to decrease at concentrations higher than 50 µM. Given that our protocol is both quick and effective, we did not further investigate variations in light treatment.

The benefit of double treatment was most evident at 1 µM PMA: while a single treatment with 1 µM PMA reduced the PCR signal of dead cells by only 6.4 ∆C_t_ (~1.7 log₁₀ GU), double treatment increased this suppression to 12.2 ∆C_t_ (~3.3 log₁₀ GU reduction). The difference between double treatment with 10 or 25 µM PMA compared to single treatment was up to 3 cycles; however, these differences were not statistically significant. In both single and double treatments with 10 or 25 µM PMA, C_t_ values were already close to the LOD. Moreover, the effect of dark incubation time was notable: by increasing the dark-exposure time from 1 min to 5 or 15 min, double treatment with 10 µM PMA completely suppressed the PCR signal from 10^7^ dead cells per sample to below the LOD.

In summary, the optimized protocol reliably detects live cell counts, even in the presence of a high background of dead cells (Fig 2), with only a slight effect on live cells.

### 4.2. Detection of *S. aureus* in food samples

Food samples are complex matrices that can affect the efficacy of vPCR. The interaction of PMA with organic and inorganic compounds, as well as a potentially high number of dead cells, may reduce the available dye concentration. Additionally, factors such as salt concentration, pH, and turbidity may further influence PMA’s interaction with DNA [23].

Our optimized protocol effectively suppressed PCR signals from dead cells to below the LOD in ground pepper, -oregano, infant milk powder, and the PW control, with minimal impact on live cells. Similar success was reported by Zhang et al. [37] when applying vPCR to detect *S. aureus* in milk powder and various meat products at an artificial contamination level of 10⁵ dead cells/g. Our contamination level of 2.7 × 10⁸ cells/ml (~10⁹ cells/g) exceeds the highest reported microbial loads in spices (up to 8 log₁₀ cfu/g of aerobic mesophilic bacteria [5,8,50]), demonstrating the robustness of the vPCR approach.

While the protocol was effective in several matrices, residual PCR signals from dead cells were detected in ground allspice, -paprika, and -pork. Turbidity, and reduced dye availability might be potential factors contributing to the incomplete signal suppression. For effective vPCR, a clear solution is crucial for photolysis. However, paprika’s red pigments and similar components in allspice cannot be removed through simple centrifugation-based washing, potentially hindering complete PMA photolysis. Furthermore, only larger particles were removed during washing, while smaller suspended particles remained and may hinder PMA penetration into the dead cells. These spice particles may also reduce available PMA molecules due to non-specific binding. A further factor that might affect the PMA-to-DNA stoichiometry is the high autochthonous microbial load in the spices used [8]. According to Codony et al. [38], 5 µM PMA theoretically contains enough molecules to neutralize 10^7^ cells/ml; however, because PMA interacts not only with DNA but also with other organic molecules in the cell and the food matrix, a higher concentration may be required. Since higher PMA concentrations negatively affect live cells, incorporating a third PMA treatment might resolve the PMA-to-DNA ratio issue.

Detection of *S. aureus* in spices and herbs using PCR is generally challenging due to the presence of PCR inhibitors in these matrices. Cabicarová et al. [13] demonstrated that a washing step with a solution containing Triton X-100 can improve detection sensitivity. Particularly when enrichment is involved, washing is necessary to reduce growth-inhibitory factors, allowing *S. aureus* to grow. While washing is necessary to reduce inhibitors and turbidity, washing can cause cell loss – especially in ground matrices with high surface areas. Bacteria attached to removed rough particles may be lost, likely contributed to the elevated C_t_ values observed for both treated and untreated live cells in spice samples. A further explanation for the elevated C_t_ values might be the presence of PCR inhibitors in the spices [13]. Incorporation of an internal amplification control is therefore recommended when working with spices.

Given that the contamination levels used in our study are likely higher than those encountered in real-world conditions, this test primarily serves as a theoretical evaluation. Therefore, we conducted an additional experiment to simulate a more realistic contamination scenario in food matrices using a mixture of 1.9 cfu/ml live cells and 4.8 × 10⁶ dead cells/ml. The aims were: (1) to assess the efficacy of the protocol in suppressing the PCR signal of 10⁶ dead cells/ml (~10^7^ cells/g), and (2) to assess the protocol’s ability to accurately detect viable cell counts that naturally increase during enrichment. Alongside vPCR, traditional plate counts on selective Baird-Parker agar were performed as a control.

With a dead cell concentration two orders of magnitude lower than in the previous experiment, our optimized vPCR protocol successfully reduced DNA amplification below the LOD (10^2^ cfu/ml). Since viable cells were inoculated below the culture-based LOD (10¹ cfu/ml), no colonies were detected on Baird Parker agar. Both vPCR and culture results were consistent despite the relatively high dead cell count of 10⁶ cell/ml.

After 24 h of enrichment, varying growth rates of live *S. aureus* cells were observed across the different matrices. The obtained C_t_ values were compared with those of the standard curves to assess accuracy. For most matrices, the C_t_ values were close to the expected values, with paprika yielding the highest accuracy (Table 3). Another study with a comparable experimental design reported accurate detection of *S. aureus* in milk using vPCR. Zi et al. [34] demonstrated reliable detection of *S. aureus* in milk inoculated with live cells ranging from 10² to 10⁶ cfu/ml, mixed with 10⁴ dead cells/ml, and pre-treated with Triton X-100 before PMA treatment. Their results also showed strong linearity between cell counts and corresponding C_t_ values. Although the dead cell counts in Zi et al.‘s study was two orders of magnitude lower than in our study, both studies highlight the high potential of vPCR for accurate and reliable *S. aureus* detection in food samples.

**Table 3:** Comparison between culture-based cell counts and the corresponding C_t_ values obtained using double PMA treatment, along with the mean values from the standard curves.

Food matrix	Mean cell count on Baird Parker plate in cfu/ml	Mean C_t_ value obtained with PMA treatment	Expected mean C_t_ value according to the standard curve using a PMA-treated cell mix (Fig 2A)	Expected mean C_t_ value according to the standard curve using untreated live cells (Fig 2 C2)
Ground Paprika	5.3 × 10^2^	37,23	38.02	37.71
Ground Pork	5.7 × 10^5^	29	28,39	27.94
Infant milk powder	6.0 × 10^4^	34,09	32,22	31.22
PW control	>1.0 × 10^6^	28,12	<24,90	<23.64

During enrichment, particularly after 48 h, a high autochthonous microbial background made it difficult to count *S. aureus* cells despite using selective plates. This issue might be mitigated if enrichment were conducted according to ISO 6888–3 (i.e., using a selective medium for sample dilution). For detection with vPCR, a high background of other species did not appear to affect our experiment, despite the use of unselective media.

Returning to the example cited in the introduction regarding the EU regulation that the concentration of coagulase-positive staphylococci in milk powder should not exceed 100 cfu/g: With our protocol, detection of *S. aureus* at a concentration of 5 × 10² cfu/ml is feasible; however, it is not sensitive enough to meet the regulatory requirement. Further sample preparation steps need to be optimized to increase sensitivity. A simple strategy to achieve this is to use a larger volume of the diluted sample and concentrate it through centrifugation. It should be noted that the applied DNA extraction approach used in our study yields a final volume of 800 µl. Using a different DNA extraction kit that provides a more concentrated eluate could also further enhance sensitivity.

Regarding the requirement that samples indicating values over 10⁵ cfu/g should be tested for staphylococcal enterotoxin [12], our procedure would be able to meet the required sensitivity. Since toxin production occurs only at high cell concentrations, a low limit of detection is not strictly necessary in these test cases. In certain instances, ISO 6888–3 is used as a detection method to confirm the absence of bacteria or to calculate the most probable number. In such scenarios, applying a vPCR after the enrichment step would reduce the time-to-result compared to subsequent cultural plating techniques.

### 4.3. Conclusion

The optimized vPCR has effectively and reliably suppressed signals from high dead cell counts of 10^7^ cells/sample in pure cultures and 10^6^ cells/ml in food samples (heat-killed) below the LOD. Therefore, our proposed vPCR protocol showed high potential to detect *S. aureus* in certain routine analysis cases (e.g., for excluding dead cells inactivated by methods affecting the membrane or lysed due to long-term storage). However, in contrast to culture-based methods, the proposed vPCR protocol needs to be adapted depending on the matrix being tested. For quantitative detection of *S. aureus*, further optimizations, such as establishing an internal PCR control, are required. Since this protocol is optimized to suppress PCR signals from high dead cell counts, it may not be suitable for use at low cell levels. To gain long-term experience regarding the robustness of the vPCR method, it is recommended to conduct traditional culture methods in parallel. For routine application, conducting a method comparison study in accordance with ISO 16140–2 [51] is recommended. Alternatively, vPCR could be used as a preliminary screening method, with only samples displaying a PCR signal undergoing further investigation through culture-based reference methods (ISO 6888–1, or -2).
